# The molecular basis of the most red-shifted allophycocyanin discovered to date

**DOI:** 10.1007/s11120-025-01160-7

**Published:** 2025-07-21

**Authors:** Min Chen, Wutunan Ma, Tiarne Mitchell

**Affiliations:** School of Life and Environmental Sciences, The University of Sydney, Sydney, Australia

**Keywords:** Allophycocyanin, Cyanobacteria, Far-red light photoacclimation, Phycobiliproteins, Red-shifted absorption, *Halomicronema hongdechloris*

## Abstract

Allophycocyanin (APC) protein subunits responsible for red-shifted phycobilisomes are induced under far-red light conditions. The ApcB2 (encoded by gene XM38_020890) and ApcD4 (encoded by gene XM38_020900) in *H. hongdechloris* are paralogous APC subunits encoded in the termed low-light photoacclimation (LoLiP) gene cluster, which are only detected from cells grown under far-red light (FRL) conditions. We examined the function of these allophycocyanin subunits using heterogeneous recombinant *E. coli* systems. The recombinant chromophorylated ApcB2 showed absorptionpeaking at 618 nm and fluorescence peaking at 642 nm and the chromophorylated ApcD4 demonstrated two absorption peaks of 618 and 676 nm and fluorescence peaks of 625 and 698 nm, respectively. Interestingly, the heterodimer of ApcB2/ApcD4 demonstrated even further FRL absorption of 728 nm and fluorescence emission peaking at 742 nm. Using ΔApcB2-W75T to replace ApcB2 for APC ab heterodimeric formation, the red-shifted absorption at 728 nm disappeared, suggesting that Trp75 of ApcB2 is essential for the heterodimer maintaining the red-shifted 728 nm spectroscopic feature. The extremely red-shifted spectroscopic properties of ApcD4/ApcB2 complexes reveal the strain-specific diversity of FRL-phycobilisomes and advance our understanding of remodelled light-harvesting complexes that capture FRL. In *H. hongdechloris*, besides the well-known Far-red light Photoacclimation (FaRLiP) gene cluster, the APC αβ heterodimer of ApcB2/ApcD4 from LoLiP gene cluster likely functions as the terminal emitter of red-shifted phycobilisomes for chlorophyll *f*-binding protein complexes. The recombinant, red-shifted APC αβ heterodimer offers a potential new class of fluorescence labels in the near-infrared spectral region.

**Supplementary Information:**

The online version contains supplementary material available at 10.1007/s11120-025-01160-7.

## Introduction

Cyanobacteria are the only prokaryotic bacterial phylum capable of carrying out oxygenic photosynthesis, similar to that of plants and algae. Compared with eukaryotic photosynthetic organisms, cyanobacteria use a range of alternative pigment-binding protein complexes, including large peripheral membrane antenna phycobilisomes (PBSs), which are not found in higher plants. The extrinsic antenna PBSs consist of two or three types of pigment-proteins (phycobiliproteins) along with several associated linkers (Bryant and Canniffe 2018). The intensively coloured phycobiliproteins are homologous protein groups containing covalently bound phycobilin chromophores that absorb visible light in the range of 500 to 650 nm (MacColl 1998). The structure and size of PBSs vary widely, and the most common structure has been described as hemidiscoidal PBSs supported by tri-cylindrical cores (Singh et al. 2015; Zheng et al. 2021; Bryant and Gisriel 2024). The phycobiliproteins are broadly grouped into three main types – phycoerythrin (PE), phycocyanin (PC), and allophycocyanin (APC) – based on the apoprotein identities, chromophore types, and their relative location in the antenna complex of PBSs. Each type of phycobiliproteins is linked to chromophores (billins) via one or two thioether bonds to cysteine residues in the protein and exhibits different absorption spectra (Scheer and Zhao 2008). Phycobiliproteins are composed of two related but distinct protein subunits, α and β. The heterodimer of αβ is assembled into a trimer (αβ)_3_ or a hexamer (αβ)_6_, the basic building block of PBS (Adir 2005). The arrangement of phycobiliproteins within PBSs reflect the energy transferring cascade: the shorter wavelength absorption components (PE and PC) are located on the peripheral side of PBS rods and longer wavelength absorption APC form the core of PBSs associated directly with chlorophyll (Chl)-binding protein complexes buried in the membrane. The APC core consists of bulk APC α and β subunits in a heterodimer (αβ) and a trimer of heterodimer (αβ)_3_, or a hexamer (αβ)_6_ disc. The APC core structure contains 2–5 cylinders, and each cylinder is made up of 4 discs and different core structures reflect the various PBS shapes (Bryant and Canniffe 2018). A specialized APC α − type subunit known as allophycocyanin-B (AP-B, also named as ApcD) place in the basal ab heterodimer resulting in two different α-type subunits, ApcA and ApcD, in APC core discs (Chang et al. 2015). Two terminal energy emitters of PBS are ApcD and core-membrane linker, LCM (also named as ApcE). ApcD is a member of the phycobiliprotein family (pfam00502), while ApcE has a more complex structure, comprising a chromophorylated domain and some additional linker repeats (pfam00427). The numbers of pfam00427 domain in ApcE determine the overall structure of PBSs and ApcE having three pfam00427 domains supports hemidiscoidal PBS shapes (Zheng et al. 2021). The excitation energies of ApcD (655 nm) and ApcE (~ 670 nm) can transfer all excitation harvested by the PBS to the Chl-binding protein complexes inside of thylakoid membranes (Gindt et al. 1992; Liu et al. 2013).

The single APC-α or APC-β has an absorption peak at around 615 nm, after formation of an α/β heterodimer, the absorption maximum of APC shifts to approximately 650 nm with a shoulder at 610 nm (Peng et al. 2014). The ApcD subunit has even further red-shifted absorption at ~ 670 nm (Dong et al. 2009) and the special ApcD subunits from Chl *f*-producing cyanobacteria exhibit the extremely red-shifted absorption maxima of ~ 712 nm (Li et al. 2016; Ho et al. 2017).

Many cyanobacteria can alter their PBS composition in response to a changing light environment in a process called chromatic acclimation (CA) in order to increase the portion of the pigment with absorption more nearly to the available light (Kehoe 2010). Seven CA variants have been classified as CA1–CA7 (Hirose et al. 2019; Sanfilippo et al. 2019; Wang and Chen 2022) and CA6 represents the assembly of far-red light (FRL) absorbing PBSs and upregulation of the far-red light photoacclimation (FaRLiP) gene cluster (Gan et al. 2014; Zhao et al. 2014; Zhang et al. 2019; Antonaru et al. 2020). The regulation of CA6 is based on photoreceptor control (Zhao et al. 2015). In the Chl *f*-producing cyanobacteria, FaRLiP is reported as a unique photoacclimation in which gene cluster contains additional photosystem reaction centre genes, Chl *f* synthase (ChlF) and ApcD subunits of red-shifted PBS (Ho et al. 2016; Li et al. 2016; Wang and Chen 2022). *Halomicronema hongdechloris* (*H. hongdechloris*) is classified as a member of CA6 group in which the red-shifted PBS are formed as bicylindrical APC core structure and made of APC subunits only under FRL conditions, but under white light (WL) conditions conventional PBS made of PC and APC (Li et al. 2016). The sequences and structure of ApcD variants from FaRLiP gene cluster lead to their changed spectral properties (Ho et al. 2017; Soulier et al. 2020; Chen et al. 2022; Gisriel et al. 2024a). When grown in FRL conditions, Chl *f*-producing cyanobacteria produces the Chl *f* and FRL absorbing PBS (FRL-PBS) to maximise the photosynthetic light harvesting capability (Bryant et al. 2020; Schmitt and Friedrich 2024). The architectures of photosynthetic membranes in *H. hongdechloris* are different in the cells grown under white light (WL) from the cells grown under FRL conditions (Li et al. 2016; Chen et al. 2019). This is a photo-reversible CA process involving newly synthesized proteins within the FaRLiP gene cluster (Li et al. 2014). The relatively increased levels of APC peptides in *H. hongdechloris* are the results of upregulated FaRLiP gene cluster, including new biosynthesis and assembly of photosystems (Chen et al. 2019). In contrast, the relatively decreased PC protein level under FRL conditions may be due to halted PC synthesis after transfer to FRL conditions, although we do not know whether the degradation of pre-existing PBSs is controlled by the same mechanisms (Chen et al. 2019). Therefore, many Chl *f*-producing cyanobacteria maintain a variable amount of WL-PBS components even after weeks of FRL acclimation (Ho et al. 2020; Mascoli et al. 2020).

Gisriel et al. resolved the structure of a red-shifted bicylindrical APC core structure using the isolated PBS core from *Synechococcus* PCC 7335 grown under FRL conditions for 2 months (Gisriel et al. 2024b). The APC subunits in FRL-PBS from *Synechococcus* PCC 7335 were arranged as 2 trimers (αβ)_3_ face to face, consistent with previous reports of the red-shifted PBS isolated from *H. hongdechloris* (Li et al. 2016; Gisriel et al. 2024b). The red-shifted PBSs are vital for efficient energy transfer to the Chl *f*-binding photosynthetic complexes embedded in the thylakoid membrane (Majumder et al. 2017; Mascoli et al. 2020; Soulier et al. 2020).

Phycocyanobilin (PCB) is the chromophore covalently attached to conserved cysteine residues in PC and APC (Silder 1994). PCB is derived from heme by heme oxygenase (HO) and ferredoxin-dependent bilin reductase (PcyA) within cyanobacteria (Dammeyer and Frankenberg-Dinkel 2008) and engineered *E. coli* containing these two enzymes produces PCB (Zhao et al. 2006). Free PCB prefers to form a cyclohelical conformation in solution and shows no photochemical properties. PCB associated with variant apo-phycobiliproteins is responsible for special spectral features of chromophorylated phycobiliproteins (holo-phycobiliproteins). The spectroscopic properties of each chromophore within phycobiliproteins are strongly affected by the conjugation and protein environment imposed on PCB (Peng et al. 2014; Tang et al. 2015). The bound PCB in ApcD2 from *Synechococcus PCC 7335* demonstrated planar configuration supporting the red-shifted absorption properties of isolated FRL-PBS (Gisriel et al. 2024b).

Most chromophore ligation to phycobiliprotein subunits occurs post-translationally and is catalysed by phycobiliprotein lyase, although spontaneous attachment of PCB with low fidelity in vitro has been reported (Fairchild and Glazer 1994). The specific bilin lyases (CpcS) catalysing PCB ligation to APC in *H. hongdechloris* were reported and the PCB binding sites on APCx in *H. hongdechloris* are chromophorylated by a minimum of two CpcS (Li and Chen 2022). There are 4 copies of *apcB* and 5 *apcD* genes annotated in the *H. hongdechloris* genome and genes *apcB1* and *apcD1-3* are inside the FaRLiP gene cluster upregulated under FRL conditions (Chen et al. 2019).

The recently reported low-light photoacclimation process (LoLiP) upregulates a three-gene cluster including isiX, ApcD4, and ApcB3 from some cyanobacteria (Olsen et al. 2015; Soulier et al. 2020, 2022). The recombinant ApcD4-ApcB3 oligomers form a helical nanotube structure with maximal absorption of 710 nm from a thermophilic *Synechococcus* sp (Gisriel et al. 2023). *H. hongdechloris* genomes includes this LoLiP gene cluster including the annotated *isiA2*, *apcB2* and *apcD4* genes, but no GAF-containing photoreceptors nearby although they were upregulated under FRL conditions (Chen et al. 2019). Here we characterised the functional role of ApcB2/ApcD4 using in vitro recombinant protein studies. We find that a heterodimer of ApcB2/ApcD4 has a signature absorption peak at 728 nm with corresponding fluorescence emission peak of 742 nm, the most red-shifted absorption of APC a/b dimer. The Trp-75 residue in ApcB2 is unique residue and is vital for formation of the 728 nm component. The replacement of cysteine residues in ApcD4 will resolve the interacting manners between PCB and the protein. This is the first report of an APC heterodimer having such red-shifted absorption, suggesting the heterodimer of ApcB2/ApcD4 functions as the terminal bridging energy transfer from FRL-PBS to the Chl *f* + *a*-binding photosynthetic complexes.

## Methods and materials

### Construction of recombinant expression plasmids

The genes *ApcD4* (XM38_020900) and *ApcB2* (XM38_020890) synthesised by Integrated DNA Technologies (IDT) with additional 5ʹ-GCA GCG GCC TGG TGC CGC GCG GCA GCC AT*A TG* and 3ʹ-*TAG* CGG CTG CTA ACA AAG CCC GAA AGG AAG CTG at the ends of gene open reading frames (ORFs). The synthesised DNA fragments of *ApcD4* and *ApcB2* were cloned into pET15b using the restriction enzymes NdeI and BamHI. The pET15b vector includes a His_6_ tag encoding region, which will be added to the N-terminus of APC for further purification of the proteins. For generating reconstituted chromophorylated APC subunits, pET15b-*ApcD4* or pET15b-*ApcB2* was transformed into *E. coli* BL21*™(DE3) (Novagen), containing pACYduet-ho1:pcyA and pCDFDuet-cpcS’s as described in Li and Chen (2022). The positive thrice-transformed *E. coli* clones were selected on Luria–Bertani (LB) plates in the presence of ampicillin (100 µg/ml), chloramphenicol (34 µg/ml), and spectinomycin (50 µg/ml). When thrice-transformed *E. coli* culture reached OD_600nm_ at the range of 0.5–0.6, 0.5 mM IPTG (isoprophyl β-D-thiogalactoside) was added and continuous cultured at 16 °C for overnight. The *E. coli* cells were centrifuged at 12,000 x g for 5 min at 4 °C and rinsed with water. The cell pellets were stored at -80 °C until required.

The site-direction mutagenesis technologies (Q5 Site-Directed Mutagenesis Kit, New England Biolabs Inc) were applied for generating four site-mutants of Cys/Ser, named as ΔApcD4-C62S, ΔApcD4-C78S, ΔApcD4-C92S, and ΔApcD4-C130S (Fig. S1). ApcB2 mutants of C16S was achieved using site-direction mutagenesis (Fig. S1). The designed special primers for site-mutagenesis are listed in Table S1. Additionally, a ΔApcB2-E67C and ΔApcB2-W75T DNA fragments were synthesised by IDT and were cloned into pET15b, then transformed into *E. coli* BL21*™(DE3) (Novagen), containing pACYduet-*ho1*:*pcyA* and pCDFDuet-*cpcS*’ as described above.

The DNA fragment containing ApcB2 and ApcD4 with a ribosome-binding site (GAAGGAGAT) between two gene open reading frames was synthesised by IDT with additional 5ʹ-GCA GCG GCC TGG TGC CGC GCG GCA GCC AT*A TG* in front of ApcB2 ORF and 3ʹ-*TAG* CGG CTG CTA ACA AAG CCC GAA AGG AAG CTG at the ends of ApcD4 ORF for co-expression ApcD4 and ApcB2. The synthesised DNA fragment was cloned into pET15b, then transformed into *E. Coli* BL21*™(DE3) (Novagen), containing pACYduet-*ho1*:*pcyA* and pCDFDuet-*cpcS*’ as described above. All expression constructs were sequenced at Garvan Institute of Medical Research (Sydney, Australia) to confirm that no unwanted mutations had been introduced.

### Recombinant APC and the mutant’s protein expression and purification

The recombinant chromophorylated APC were purified by standard methods as described in Li and Chen (2022). Briefly, cell pellets were resuspended in lysis buffer (20 mM potassium phosphate buffer, pH 7.6, 0.5 M NaCl, 5 mM imidazole) and disrupted by 3 cycles of Fast-Prep (MP Biomedicals). The resulted lysate was pelleted by centrifugation16,000 x g for 20 min at 4 °C. The supernatant was loaded onto a Ni-NTA column (His GraviTrap 1 ml, GE healthcare-Life Sciences) and developed with the 5-column volume of start-buffer containing 10 mM imidazole and rinsed in the same buffer (20 mM potassium phosphate buffer, pH 7.6, 0.5 M NaCl, 10 mM imidazole). The His-tagged protein was eluted in elution buffer (20 mM potassium phosphate buffer, pH 7.6, 0.5 M NaCl, 0.5 M imidazole). The purified protein concentration was estimated by absorption reading at ~ 280 nm. Protein electrophoresis was performed on 4–15% gradient or 12% pre-cast Bolt gels (Thermo Fisher Scientific). The chromophore-carrying proteins were visualised by Zn^2+^ induced fluorescence and total proteins were stained using InstantBlue^®^ (Abcam).

### Chromatographic purification and analysis

To characterise recombinant PCB-binding APC proteins, the interested holo-proteins were further purified by size-exclusive chromatography SEC70 (Enrich™ SEC70 10 × 300 mm, Bio-Rad) using buffer of 40 mM potassium phosphate buffer, pH 7.4, 50 mM NaCl and 5% (w/v) glycerol at flow rate of 0.5 ml/min on FPLC (NGC Chromatography System, Biorad). Some interested holo-protein samples were also further analysed by BioSpec-SEC3000 (300 × 7.8 mm, 3.0 μm particle size, Phenomenex) using 40 mM potassium phosphate and 50 mM NaCl buffer, pH 7.4 at the flow rate of 1.0 ml/min on uHPLC (Nexera system, Shimadzu).

### Spectral analysis

Absorption spectral analyses were performed using a Shimadzu UV–VIS 2550 spectrophotometer. Steady-state fluorescence spectral analysis was performed using a Varian Cary Eclipse Fluorescence spectrophotometer. The red- and far-red fluorescence of recombinant holo-protein of PCB-binding APC was monitored using excitation wavelength at 580 nm at RT except as noted below. To increase the signal-to-noise ratio, the fluorescence spectra were obtained from the average of 10 repeated readings and the samples were diluted to a maximum absorbance < 0.1 in the range of 400–750 nm using the same buffer.

## Results

### In silico analysis of ApcD4 and ApcB2 from *H. hongdechloris*

In *H. hongdechloris*, encoding genes *ApcD4* (474 bp, XM38_020900) and *ApcB2* (483 bp, XM38_020890) are additional APC paralogous in LoLiP gene cluster, whose transcript levels increases in cell grown under FRL conditions (Chen et al. 2019). These two proteins are contributed for red-shifted PBS (Li et al. 2016).

**Fig. 1:**
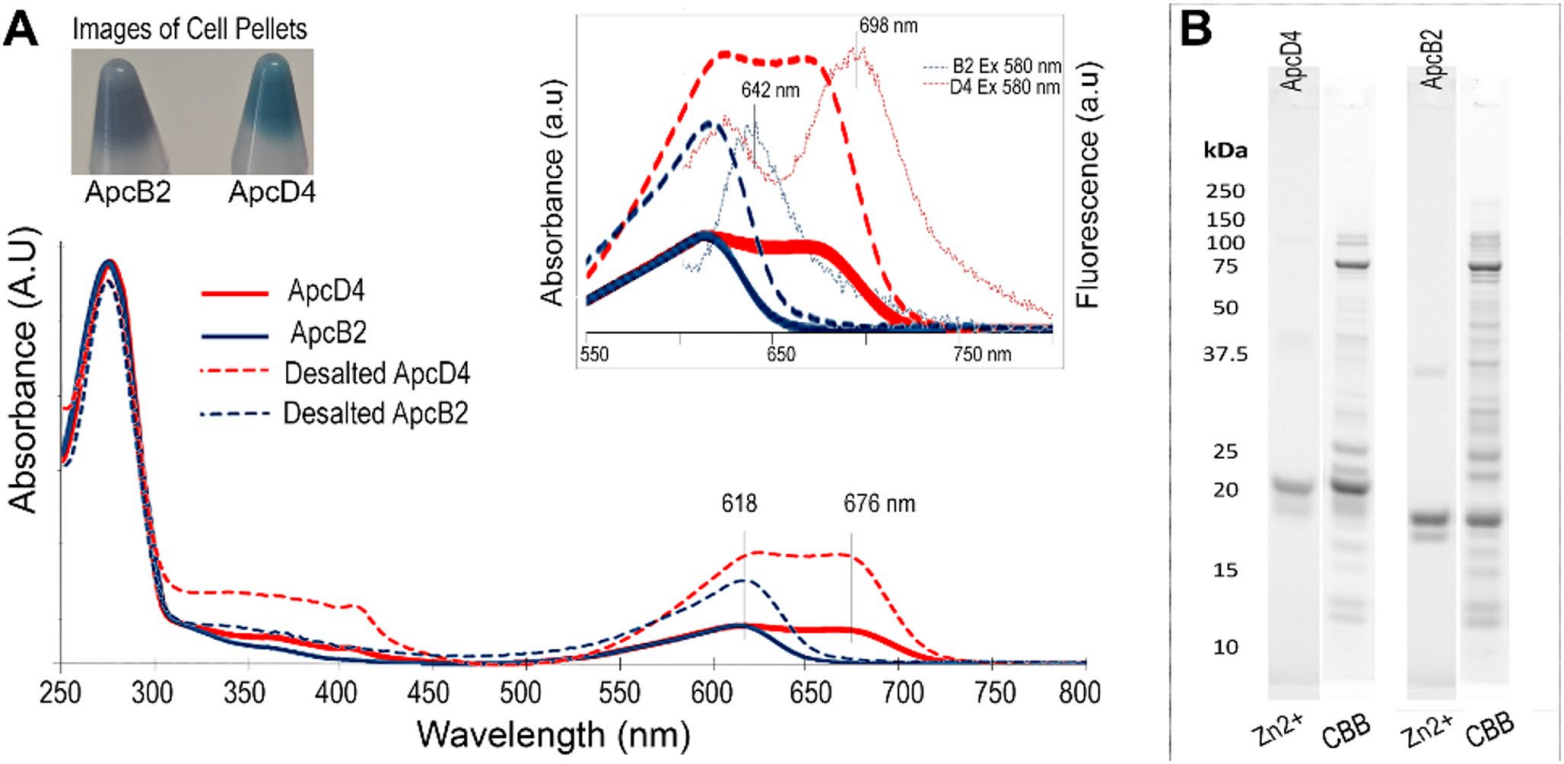
Spectra and protein electrophoresis of PCB-ApcB2 and PCB-ApcD4. (**A**) Absorption spectra recorded in the elution buffer containing 0.5 M imidazole (solid lines), in desalting buffer containing 40 mM potassium phosphate and 50 mM NaCl (dashed lines), and emission fluorescence (thin lines) were excited at 580 nm (in A insert). (**B**) Protein gel visualised by Zn^2+^-induced fluorescence and CBB staining (InstantBlue^®^). All samples were purified by Ni^2+^ affinity chromatography. All spectra were normalised at 280 nm. Red lines, PCB-ApcD4 samples; Blue lines, PCB-ApcB2 samples

We examined the roles of ApcD4 and ApcB2 in a heterologous system where ApcD4 and ApcB2 were co-expressed with pACYduet-*ho1:pcyA* and pCDFDuet-*cpcS*’s plasmids in *E. coli* to produce PCB binding-ApcD4 and PCB binding-ApcB2, respectively. Hexahistidine-tagged (His_6_)-ApcD4 and (His_6_)-ApcB2 was purified using Ni-NTA affinity chromatography after cell lysis and PCB binding-ApcD4 showed two main absorption peaks centred at 618 nm and 676 nm (Fig. 1). Isolated chromophorylated ApcB2 showed an absorption peak of 618 nm. Both chromophorylated ApcD4 and ApcB2 maintained their absorption profiles after removing 0.5 M imidazole (Fig. 1). Isolated chromophorylated ApcD4 showed fluorescence emission peaks of 625 and 698 nm and chromophorylated ApcB2 had a fluorescence emission at 642 nm using 580 nm excitation at room temperature (Fig. 1 insert). The fluorescence excitation spectra further denoted that 676 nm is the main PCB-ApcD4 absorption component and 618 nm component is the main component from PCB-ApcB2 (Fig. S2). The covalent attachment of PCB in chromophorylated ApcD4 and chromophorylated ApcB2 were verified by Zn^2+^-enhanced fluorescence (Fig. 1B). The visualised ~ 38 kDa band were assigned as dimeric of ApcD4 and ApcB2, respectively (Fig. 1B).

The PCB-ApcD4 was further purified by BioSep-SEC3000 (Phenomenex) on uHPLC using 40 mM phosphate buffer as mobile phase at flow rate of 1 ml min^− 1^ and the main elution was collected at approximately 12 min, corresponding molecular weight of ~ 20 kDa. The purified PCB-ApcD4 showed absorption peaks of 618 and 676 nm, consistent with spectral profiles of chromophorylated ApcD4 (Fig. S3). Using 8 M urea to denature chromophorylated ApcD4 in 40 mM phosphate buffer, pH 7.0 and PCB in denatured chromophorylated ApcD4 was observed (Fig. S3C).

**Fig. 2:**
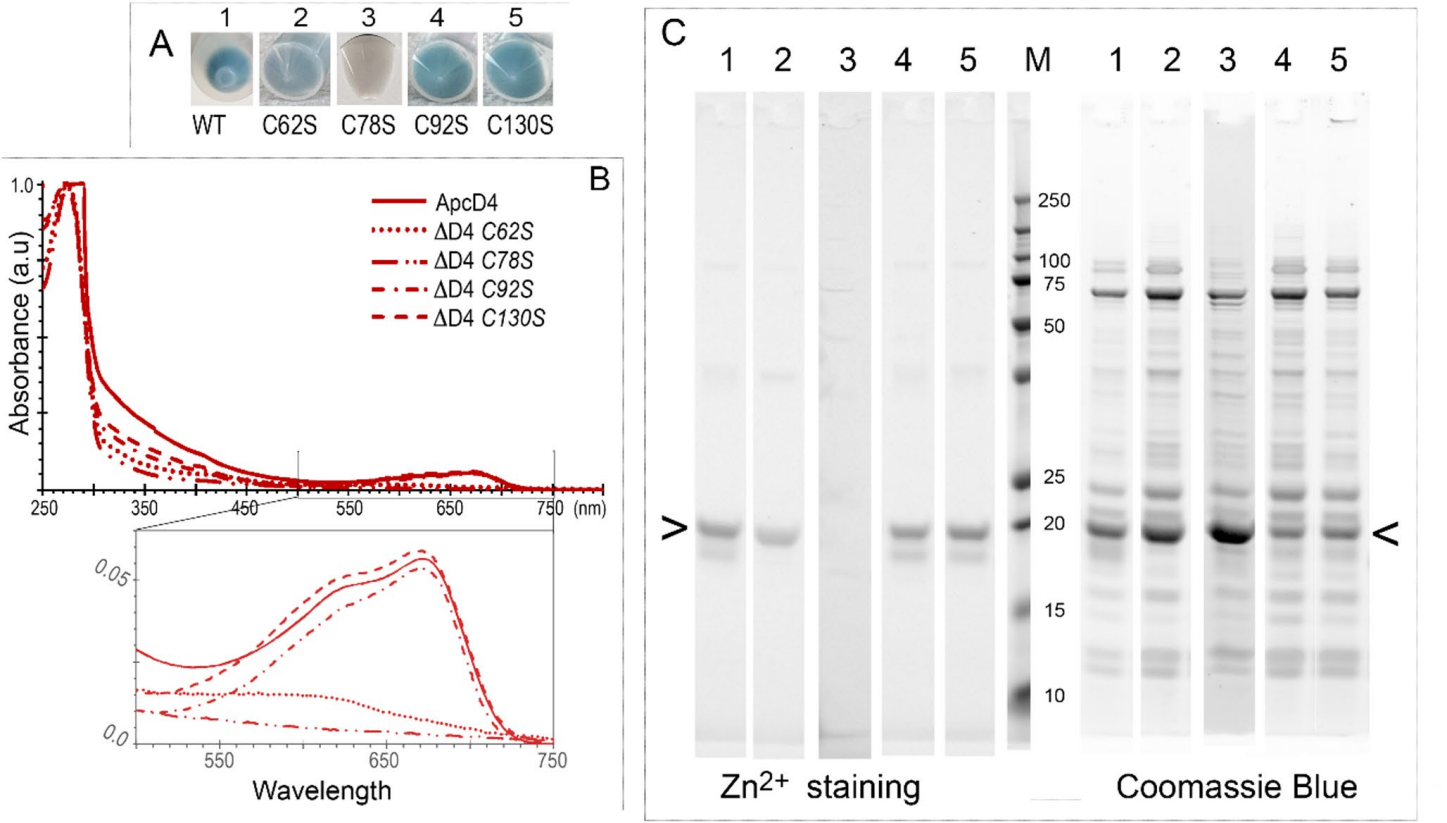
Spectra and protein electrophoresis of ApcD4 and ApcD4 mutants. (**A**) *E. coli* cell pellets containing recombinant ApcD4 proteins. (**B**) Absorption spectra recorded in the elution buffer containing 0.5 M imidazole and the detailed spectral profiles in the range of 500–750 nm (B insert). (**C**) Protein gel visualised by Zn^2+^-induced fluorescence and CBB staining (InstantBlue^®^). All samples were purified by Ni^2+^ affinity chromatography. Solid lines, Wild type ApcD4; Dotted line, ΔApcD4-C62S samples; Dash-dot-dot lines, ΔApcD4-C78S samples; Dash-dot lines, ΔApcD4-C92S samples; dashed lines, ΔApcD4-C130S samples. Arrow (< ) indicates ApcD4 protein and its mutants. All spectra were normalised at 280 nm

There are 4 Cys amino acids in ApcD4. Besides the conserved Cys78, Cys are positioned at sites of 62, 92 and 130 (Fig. S1). To further examine PCB attachment on these Cys sites in ApcD4 a series of site-mutants of Cys/Ser were co-expressed with pACYduet-*ho1:pcyA* and pCDFDuet-*cpcS*’s plasmids to produce chromophorylated ApcD4 mutants. The mutant ΔApcD4-C78S displayed no colour in the cell pellets and absorption of isolated His_6_-ΔApcD4-C78S confirmed no absorbances in the ranges of 500–750 nm (Fig. 2). Hence, ΔApcD4-C78S mutant eliminated the capability of binding PCB. Both ΔApcD4-C92S and ΔApcD4-C130S showed blue coloured cell pellets and the similar absorption spectra as wild-type (WT)-ApcD4 (Fig. 2). Unexpectedly, cell pellets of ΔApcD4-C62S showed blueish colours, but with decreased and changed absorption profiles (Fig. 2B insert). ΔApcD4-C62S displayed no detectable fluorescence emission peaks in the range of 500–750 nm (Fig. S4) although the Zn^2+^ induced fluorescence confirmed covalently bound PCB in isolated ΔApcD4-C62S (Fig. 2C). The replacement of Cys62 may disrupt the PCB attachment to Cys78 partially, result into unexpected interactions between PCB and protein, or possibly make ApcD4 unable to fold. Mutants ΔApcD4-C92S and ΔApcD4-C130S demonstrated enhanced fluorescence emission at ~ 698 nm using excitation of 580 nm (Fig. S4).

**Fig. 3:**
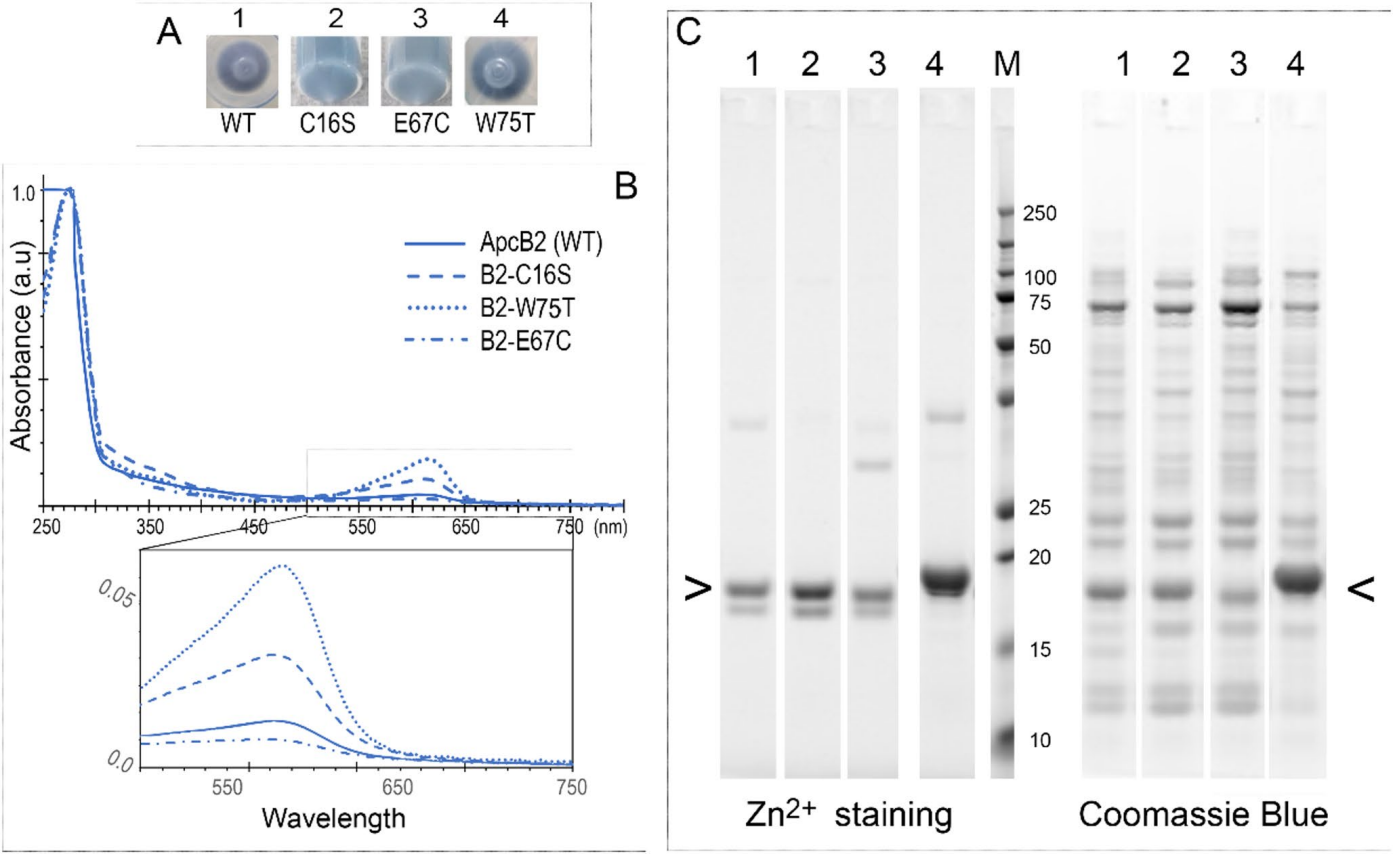
Spectra and protein electrophoresis of ApcB2 and ApcB2 mutants. (**A**) *E. coli* cell pellets containing recombinant ApcB2 proteins. (**B**) Absorption spectra recorded in the elution buffer containing 0.5 M imidazole and detailed spectral profiles in the range of 500–750 nm (B insert). (**C**) Protein gel visualised by Zn^2+^-induced fluorescence and CBB staining (InstantBlue^®^). All samples were purified by Ni^2+^ affinity chromatography. Solid lines, Wild type ApcB2; dashed line, ΔApcB2-C16S samples; Dotted lines, ΔApcB2-W75T samples; Dash-dot lines, ΔApcB2-E67C samples. Arrow (<) indicates ApcB2 protein and its mutants. All spectra were normalised at 280 nm

There are two Cys residues in ApcB2. Besides the conserved chromophore binding Cys at position 81, a second Cys is located at the position 16 (Fig. S1). The mutant of ΔApcB2-C16S displayed no changes for ApcB2 chromophorylation (Fig. 3). Following sequence alignment, Glu67 and Trp75 in ApcB2 are different from others (Fig. S1B). The ΔApcB2-W75T and DApcB2-E67C displayed chromophorylated contours as WT-ApcB2, an absorption peak at 618 nm (Fig. 3B insert). However, the mutant E67C exhibits low PCB absorbance and fluorescence emission (Fig. S4), suggesting it was only partial ΔApcB2-E67C chromophorylated.

### Formation of recombinant heterodimer of ApcD4 and ApcB2

**Fig. 4:**
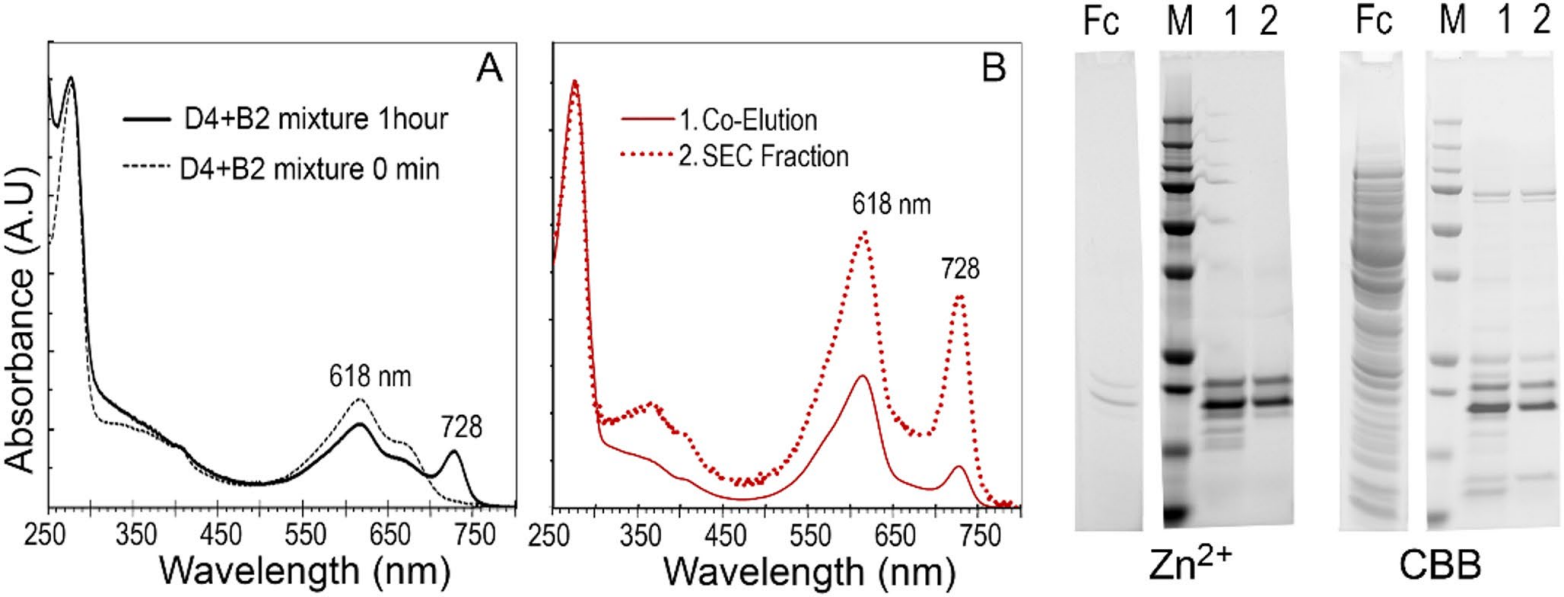
Evidence of 728 nm component formation. (**A**) Compared spectra of a mixture of purified ApcB2 and ApcD4 (0 min, dotted line) and after incubation 1 h (solid line). (**B**) ApcD4 and ApcB2 *E coli* lysate were mixed at 1:1 ratio, then loading on Ni^2+^ affinity column for co-elution (solid line). Then the sample was further purified by SEC70 gel filtration (Dotted lines). Spectra were recorded in the elution buffers (1, in buffer containing 0.5 M imidazole; 2, in 40 mM potassium phosphate buffer). C, Protein gel visualised by Zn^2+^-induced fluorescence and CBB staining (InstantBlue^®^). Fc, Ni^2+^ affinity column co-elution flowthrough; 1, Ni^2+^ affinity column co-elution sample; 2, further SEC70 (Bio-Rad) purified sample; M, molecular marker. All spectra were normalised at 280 nm

**Fig. 5:**
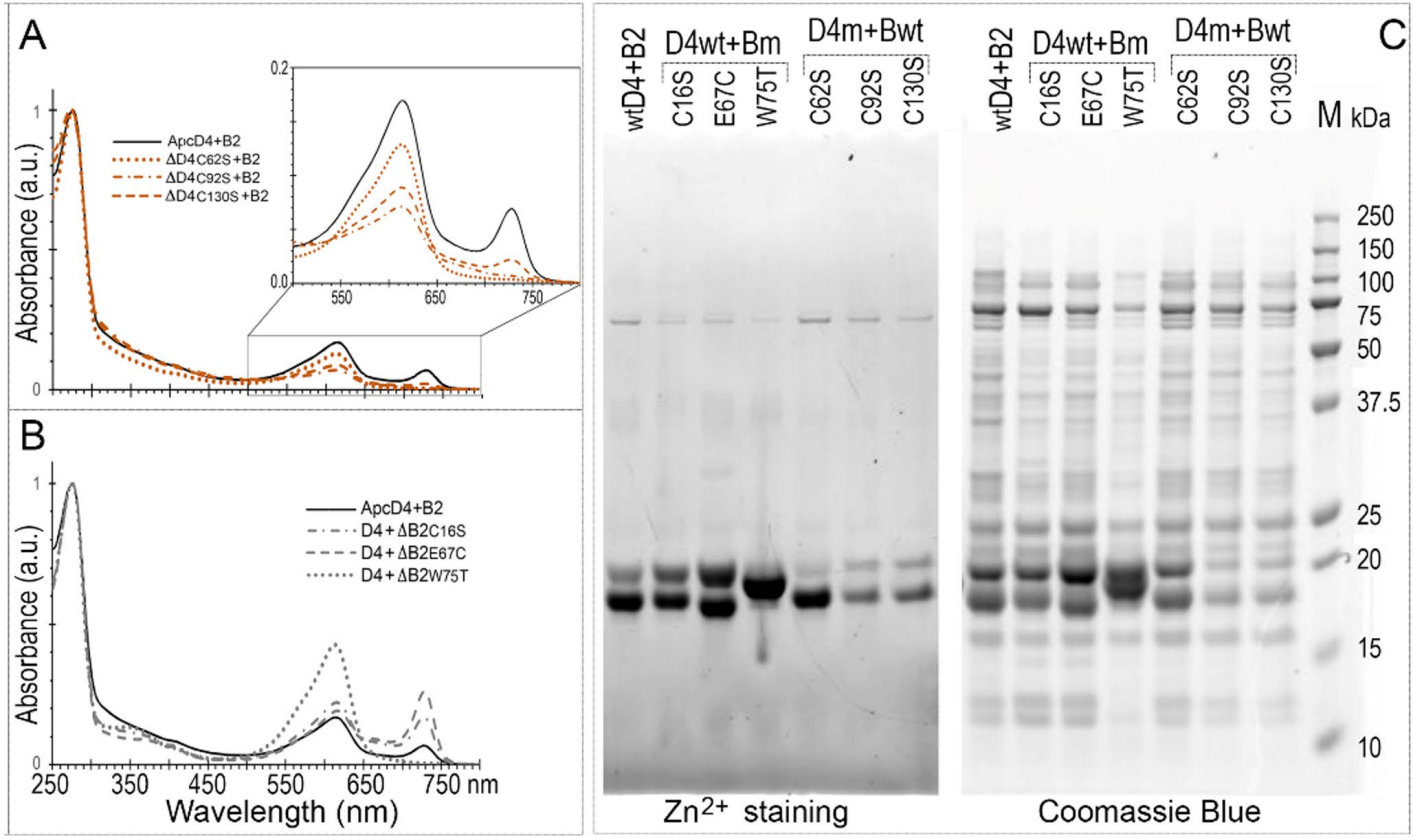
Site-directed mutants’ impact on 728 nm component formation. (**A**) Spectral comparison of ApcB2 (B2) mixed with ApcD4 (Solid black line) and ApcD4 mutants. Dotted line, ApcB2 with ΔApcD4-C62S (C62S); dash-dot line, ApcB2 with ΔApcD4-C92S (C92S); dashed line, ApcB2 with ΔApcD4-C130S (C130S). The detailed spectroscopic features in the range of 500–800 nm were presented as A-insert. (**B**) Spectral comparison of ApcD4 (D4) with ApcB2 (solid black line) and ApcB2 mutants. Dash-dot line, ApcD4 with ΔApcB2-C16S (C16S); dashed line, ApcD4 with ΔApcB2-E67C (E67C); Dotted line, ApcD4 with ΔApcB2-W75T (W75T). (**C** Protein gels were visualized by Zn^2+^ induced fluorescence and Coomassie Blue (InstantBlue^®^). D4wt + Bm, represents wtApcD4 mixed with ApcB2 mutants, respectively; D4m + B2, represents wtApcB2 mixed with ApcD4 mutants, respectively; M, molecular marker

The mixture of PCB-ApcD4 and PCB-ApcB2 demonstrated spectral shifts, a 728 nm absorption component is observed after 1 h incubation indicating the interaction of ApcD4/ApcB2 (Fig. 4A, Fig. S5). To enhance the product of 728 nm component, the mixed cell lysate of ApcD4 and ApcB2 was loaded on to Ni-NTA affinity column to collect heterodimer of ApcD4/ApcB2 (Fig. 4B). Co-elution from the mixed lysate delivered the same product with a 728 nm component and further purification by gel filtration (SEC70) verified that the heterodimer of ApcD4/ApcB2 is relatively stable and is responsible for generating the shifted spectroscopic properties (Fig. 4, Fig. S6). The protein gel verified all collected fractions having an equal amount of ApcD4 and ApcB2, but the ApcD4 in fraction 5 had lower resolution following Zn^2+^ induced fluorescence visibility (Fig. S6C). The variant configuration of attached PCB and the possible inconsistent protein folds made in the recombinant ApcD4 could be the reasons for several unsuccessful attempts to crystallizing the 728 nm component. To determine the potential mechanism of formation of such a red-shifted absorption peak, the mixture of WT-ApcB2 with ApcD4 mutants at 1:1 ratio (equal at 280 nm reading) was prepared. The mixture of ApcB2 and ΔApcD4-C130S displayed the formation of the 728 nm component. However, neither mixtures of ApcB2/ΔApcD4-C61S nor ApcB2/ΔApcD4-C92S exhibited the formation of the 728 nm component (Fig. 5A), indicating that Cys61 and Cys92 play important roles for the formation of the 728 nm component. The protein gel corroborated that the mixtures contain approximately a 1:1 ratio and ApcD4 mutants showed the reduced covalently bound PCB per protein or the partial chromophorylation according to the lower resolution of Zn^2+^ induced fluorescence displayed on the gel (Fig. 5C).

Incubation of WT-ApcD4 with ApcB2 mutants together, mixtures of ApcD4/ΔApcB2-C16S and ApcD4/ΔApcB2-E67C exhibited the formation of the 728 nm component and the mutant of E67C showed increased products of 728 nm components (Fig. 5B). Unexpectedly, a mixture of ApcD4/ΔApcB2-W75T eliminated the formation of the 728 nm component, indicating that W75 from ApcB2 is responsible for the formation of 728 nm (Fig. 5).

**Fig. 6:**
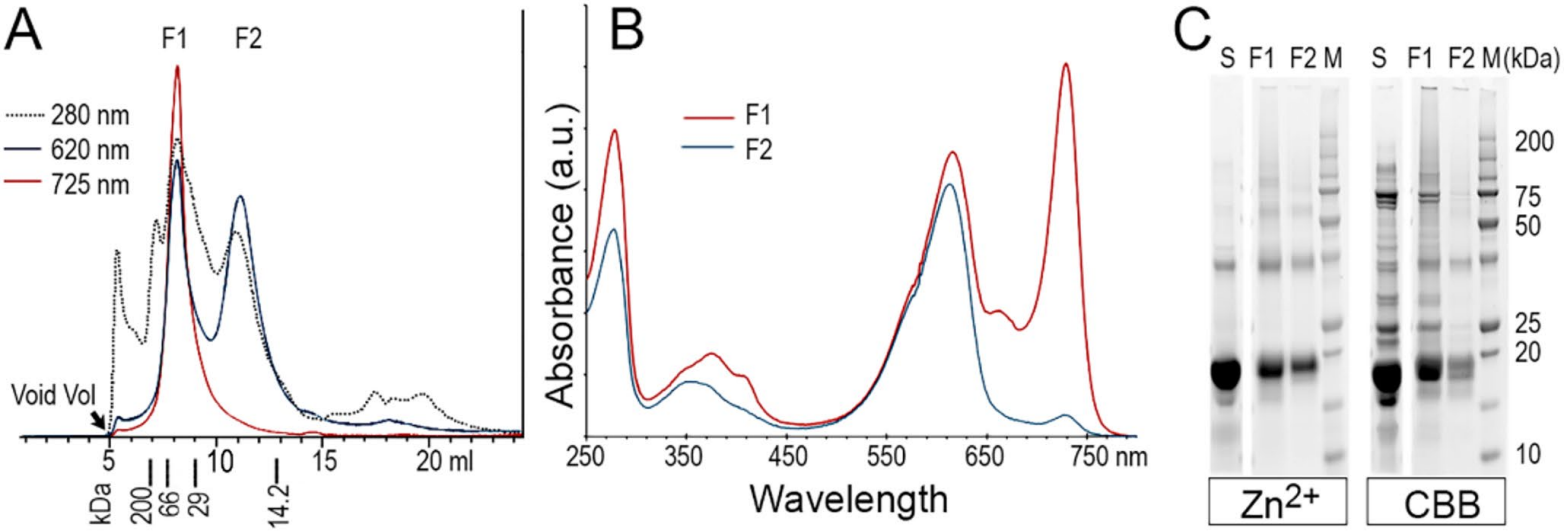
The APC ab heterodimer produced using recombinant His_6_-ApcB2 + ApcD4 heterologous *E. coli* clones. (**A**) Purification of chromophorylated APC αβ heterodimer using BioSep-SEC3000 on uHPLC. The molecular weight markers used for calibration (gel filtration markers kit, Sigma, #MWGF200-1 KT). (**B**) Online spectral comparison of resolved fractions, F1 and F2 in buffer containing 40 mM potassium phosphate and 50 mM NaCl, pH 7.4. (**C**) Protein gels were visualized by Zn^2+^ induced fluorescence and Coomassie Blue (CBB, InstantBlue^®^). S, sample prior BioSep-SEC3000 purification; F1 and F2, collected separated fractions; M, molecular marker

Attempting to increase the amount of stable heterodimer of ApcD4/ApcB2, co-expressing (His_6_)-ApcB2 + ApcD4 with pACYduet-*ho1:pcyA* and pCDFDuet-*cpcS*’s plasmids in *E. coli* were engineered. The dimer of (His_6_)-ApcB2/ApcD4 was purified using Ni-NTA affinity chromatography following the targeted (His_6_)-ApcB2 after cell lysis. The main elution (E2) indicated the 728 nm absorption feature of heterodimer of ApcB2/ApcD4 and western blotting demonstrated one positive band as expected against anti-His antibody (Fig. S7). There was a protein band at approximately 38 kDa positive against anti-His_6_, proposing to be the dimer of ApcB2/ApcD4 (star in Fig. S7B). With three individual experiments, isolated heterodimers of His_6_-ApcB2/ApcD4 showed 618 nm and 728 nm absorption peaks and PCB attachment was verified by Zn^2+^ induced fluorescence (Fig. S7C). The protein gel also verified the protein bands at about 38 kDa with positive reaction after Zn-enhanced fluorescence (Fig. S7C). Further purification using BioSep-SEC3000 gel filtration, the fraction (F1) collected at ~ 7.8 min, corresponding to molecular marker ~ 40 kDa, demonstrated the 728 nm absorption feature (Fig. 6). In contrast, the fraction (F2) collected at ~ 12 min showed dominant absorption peak of 618 nm (Fig. 6B), although the protein gel demonstrated a similar polypeptides profile (Fig. 6C).

## Discussion

The recombinant ApcB2 and ApcD4 in the engineered *E. coli* containing three plasmids are chromophorylated and resulted in blue-coloured *E. coli* cells. PCB-ApcB2 displayed conventional absorption peak at ~ 618 nm that is consistent with previously reported ApcB1 and ApcB3 from *H. hongdechloris* (Li and Chen 2022). The ApcB subunits and ApcA (XM38_033080) contain one conserved Cys81 and their monomer display an absorption peak at ~ 618 nm using the engineered *E. coli* system in our study (Fig. S8; Li and Chen 2022). Two absorption peaks in the chromophorylated ApcD4 suggest possible multiple PCB attachments or heterogenic isomers of PCB-ApcD4. ΔApcD4-C62S and ΔApcD4-C78S impaired PCB attachment (Fig. 2). Both Cys residues are conserved in ApcDs in FaRLiP and LoLiP gene clusters, except FaRLiP ApcD3 from *Synechococcus* PCC 7335 and corresponding FaRLiP-ApcD1 from *H. hongdechloris*. Both lack conserved Cys78 but have Cys62 in the BE loop region (Ho et al. 2017; Li and Chen 2022; Gisriel et al. 2024b). According to the reported structures of FRL-PBS and protein alignments, FaRLiP-ApcDs and LoLiP-ApcD4 contain the conserved short BE loop constraining the chromophore of the ApcDs (Fig. S8Li and Chen 2022; Gisriel et al. 2024a). Cys62 of ApcD4 is a residue near the pyrrole ring A with a proposed distance of ~ 8Å from the attached PCB, it might cause a tighter PCB binding pocket and disrupt the position of BE loop although Cys62 is not in the BE loop (Gisriel et al. 2024a). The replacement of Cys62 changed absorption spectral profiles, if there were chromophorylated ΔApcD4-C62S, the weaker absorbance peak of 676 nm was observed comparing with ‘double peaked’ absorption of chromophorylated ApcD4 (Fig. 2B). However, we do not know whether it is involved in covalently bound PCB or H-bonding interactions with the attached PCB (Gisriel et al. 2023, 2024b). Previously reported chromophorylated ApcD3 has the absorption peaks of 640 nm and 684 nm, similar to the chromophorylated ApcD4 in the current study (Li and Chen 2022). The two overlayered elution peaks after BioSep-SEC3000 purification might suggest co-existing isomers of PCB-ApcD4 (Fig. S3). Both ApcD3 and ApcD4 have the conserved Cys62 and Cys78, suggesting that these Cys residues associate with the attachment of PCB. In contrast, the canonical ApcD (ApcD5, XM38_031490) has only one conserved Cys78 and BE loop extended by three Aa residues compared with FaRLiP-ApcDs and demonstrated single absorption peak (Fig. S8; Li and Chen. 2022). Additionally, all paralogous ApcDs contain the conserved Trp84 residues (Fig. S8B), which could interact with the pyrrole ring D of PCB and cause red-shifted absorption (Peng et al. 2014; Kimber 2024) However, we could not resolve the one or two PCB ligated or potential multiple isomer PCB-ApcD4 in current study because we were unable to define the PCB ligation sites using peptide mass fingerprinting analysis (MALDITOF).

In the current study, we revealed that the heterodimer of ApcB2 and ApcD4 appears to have a signature absorption peak of 728 nm, the most red-shifted absorption from APC subunits. The formation of 728 nm component reached to its maximal after incubation ApcB2 and ApcD4 together for 4 h (Fig. S5). Using APC subunits from FaRLiP to replace either ApcB2 or ApcD4, no 728 nm spectral component was detected, suggesting that 728 nm component is from a heterodimer of ApcB2 and ApcD4 (Data not shown). The ΔApcD4-C92S demonstrates no impact on chromophorylation (Fig. 2), but mixtures of ApcB2/ΔApcD4-C92S were deficient in the formation of the 728 nm component. This implies that Cys92 plays an important role in the red-shifted spectral properties of bound PCB in the heterodimer of ApcB2/ApcD4. The lack of Cys92 in ApcD3 (Fig. S8) provides additional evidence for the relationship between the function of Cys92 and formation of the 728 nm component, although Cys92 was proposed to have a distance more than 11Å from the attached PCB according to the model in Gisriel et al. (2024a), an unknown mechanism for a potential long distant protein disruption on chromophore(Gisriel et al. 2024a).

There are four ApcB paralogous genes (*apcB1*–*apcB4*) and ApcB3 and ApcB4 are dominate for WL-PBS (Fig. S9). The protein alignment of ApcBs (Fig. S8A) demonstrated higher conserved homologous between ApcB in WL-PBS and FRL-PBS (Fig. S9). According to structures of FRL-APC, amino acid residues near chromophores are conserved among APC β subunits from *H. hongdechloris* (Fig. S8A). However, the conserved β subunits Thr75 of ApcB3 and ApcB4 are replaced by Trp75 in ApcB2 (current study) and Cys75 in ApcB1 (Fig. S8A). If Thr74 and Thr 75 of β subunit near the pyrrole ring D of α subunit PCB as described in Gisriel et al. (2024a), the Trp75 of ApcB2 in *H. hongdechloris* could change the interaction between pyrrole ring D of PCB bound to ApcD4 in ab heterodimer of ApcB2/ApcD4. ΔApcB2-W75T showed the same spectral profiles as WT-ApcB2, but the mixtures of ΔApcB2-W75T/ApcD4 were deficient in the formation of the 728 nm component due to changed protein environment imposing on the configuration of chromophore. The replacement of Trp75 of ApcB2 rather than the common residue of Thr75 is essential for the red-shifted spectral feature of bound PCB in the heterodimer of APC α/β heterodimer (Fig. 4). The ΔApcB2-E67C reduced chromophorylation and displayed lower amount of PCB attached (Fig. 3). The E67 of ApcB2 is a few residues away from the conserved Asn71 (Fig. S1B), which was methylated in previous reported Apc β subunits (Fig. 2 in Gisriel et al. 2024a), although we do not know whether the Asn71 was methylated in the recombinant protein. However, the replacement of E67C did not disrupt the formation of 728 nm component, a relative higher 728 component were observed after ΔApcB2-E67C interacting with ApcD4 (Fig. 5B). Using a series of Cys/Ser mutants, additional Cys16 in ApcB2 does not influence chromophore ligation and the formation of APC α/β heterodimer in vitro. Cys62 and Cys 78 in ApcD4 directly bear on chromophore ligation, suggesting they are the residues providing thioether linkages and PCB binding docket in ApcD4. The Cys92 replacement does not affect in vitro chromophorylation, but chromophorylated ΔApcD4-C92S prevented the formation of the 728 nm component suggesting Cys92 is in the place associated with interaction of ApcB2 and ApcD4.

The spectroscopic properties of each bound chromophore in phycobiliproteins are strongly influenced by the conjugation and environment imposed on the chromophores by the proteins. The red-shifted absorbance of ApcD from *Synechocystis PCC 6803* has been accredited to the co-planarity structure of the bound PCB inside of APC α subunit (Peng et al. 2014). The shorter BE loop region of FaRLiP-ApcDs and LoLiP-ApcD resulted that pyrrole ring A of PCB extent to more coplanar (Gisriel et al. 2023, 2024a). Interaction of ab subunits might make an important spectroscopic contribution, e.g. the heterodimeric formation is essential for the extremely red-shifted 728 nm formation in the current study. ApcD4 and ApcB2 of *H. hongdechloris* are in the annotated LoLiP gene cluster but without LcyA or similar GAF-containing photoregulators (Chen et al. 2019; Soulier et al. 2022). ApcD4 and ApcB2 demonstrated the similar protein levels under FRL conditions (Fig. S9; Chen et al. 2019). If ApcD4 and ApcB2 from *H. hongdechloris* formed the helical nanotube structure as reported in LoLiP-FR-AP from a thermophilic *Synthechococcus* sp, the influences of Cys92 of ApcD4 and Trp75 of ApcB2 on the formation of the 728 nm component could be due to their contribution at the interface of the APC αβ heterodimer although the experimental results supported the 728 nm component featured the formation of heterodimeric ApcB2/ApcD4 as confirmed in Fig. 4. The non-covalent binding PCB in ApcE2 from *Synechococcus PCC 7335* demonstrated another mechanism for red-shift spectroscopic properties (Miao et al. 2016). With protein purification and electrophoresis analysis, the spectroscopic observations are recorded from covalent bound PCB-proteins, excluding the possibility of shifted spectra from non-covalently bound PCB.

In summary, the red-shifted spectra of heterodimers of ApcB2/ApcD4 can be due to several factors. One possible factor for inducing such a red-shifted spectroscopic property is modified PCB-binding protein environments that change the extent of coplanar bound chromophores. The site-directed mutants ΔApcB2-W75T revealed that Trp75 of ApcB2 is vital for changed spectroscopic properties of heterodimers of ApcB2 and ApcD4, suggesting that Trp75 residues could be attributed to the modified PCB pocket of ApcD4 at the inter-ab position. The interaction between Trp75 and bound PCB in the α subunit (ApcD4) might provide a tighter chromophore pocket to change the configuration of PCB. Further detailed structural studies of these constructions could permit the resolution of this question. This work reports the unique spectroscopic properties of APC due to changed amino acid residues in the proximal interface of the ab heterodimer, providing a molecular basis to a novel class of bio-labels with absorption and fluorescence in the FRL to the near-infrared spectral region.

## Electronic supplementary material

Below is the link to the electronic supplementary material.

Supplementary Material 1

## Data Availability

No datasets were generated or analysed during the current study.

## Abbreviations

APC: Allophycocyanin
CA: Chromatic acclimation
Chl: Chlorophyll
ChlF: Chl f synthase
CpcS: Specific bilin lyases
FaRLiP: The far-red light photoacclimation
FRL: Far-red light
HO: Heme oxygenase
LoLiP: The low-light photoacclimation
PBS: Phycobilisome
PC: Phycocyanin
PCB: Phycocyanobilin
PcyA: Ferredoxin-dependent bilin reductase
PE: Phycoerythrin
WL: White light
