# Correction to: The molecular basis of the most red-shifted allophycocyanin discovered to date

**DOI:** 10.1007/s11120-025-01164-3

**Published:** 2025-08-06

**Authors:** Min Chen, Wutunan Ma, Tiarne Mitchell

**Affiliations:** School of Life and Environmental Sciences, The University of Sydney, Sydney, Australia

**Photosynthesis Research (2025) 163:40**

10.1007/s11120-025-01160-7

The original article has been corrected. In this article the author’s name Tiarne Mitchell was incorrectly written as Tiarne Michelle.

