# Supplementary material for: The molecular basis of the most red-shifted allophycocyanin discovered to date
Source: Photosynth Res. 2025 Jul 21;163(4):40. doi: 10.1007/s11120-025-01160-7 (PMC12279586; doi:10.1007/s11120-025-01160-7)
Supplement: Supplementary file 1 — Supplementary Material 1 [file 11120_2025_1160_MOESM1_ESM.pdf]

Supplementary information

1. Supplementary Table

Table S1. Primers for site-direction mutagenesis

Mutation sites	Primer names	Sequence information
ApcD4 Cys62Ser	Q5_APCD4_62_F	TGGGCTCAGTCTCCGGACGCC
	Q5_APCD4_62_R	GTAACGCTTGCTGCCTTCATC
ApcD4 Cys78Ser	Q5_APCD4_78_F	ACTGCTAATTCCCAACGCGATC
	Q5_APCD4_78_R	TTTTTGAGGGTCGCCGCT
ApcD4 Cys92Ser	Q5_APCD4_92_F	ATAAGTTACTCCGTTTTGGCAG
	Q5_APCD4_92_R	AATACGGATGTACCAGCC
ApcD4 Cys130Ser	Q5_APCD4_130_F	GCATATCGTTCCATCAAGGAGG
	Q5_APCD4_130_R	CACCTTGTGGTACGACAG
ApcB2 Cys16Ser	Q5_ApcB3_M16_F	GATGAGCGATCTGCTTACTTGGAAGATTCCAG
	Q5_ApcB3_M16_R	AGCGGGGTTAATGGCGGC

2. Supplementary Figures

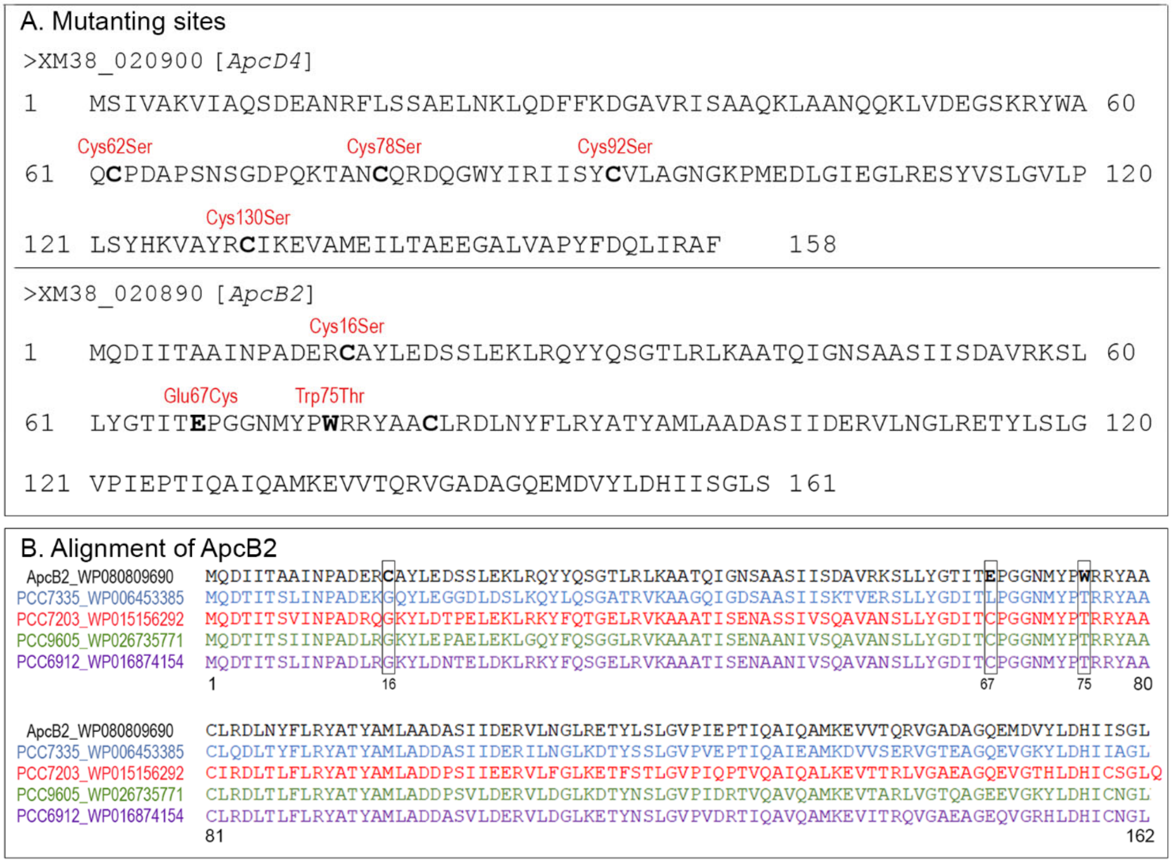

Figure S1. Protein sequence information for site mutants. A. The position of site mutants from ApcD4 (XM38_020900) and ApcB2 (XM38_020890). B. ApcB2 and selected homologous protein sequence pile-up alignment to identify the species-specific residues. PCC7335, *Synechococcus* sp. PCC 7335 (annotated as ApcB3, Gisriel *et al.* 2024b); PCC7203, *Chroococcidiopsis thermalis* PCC 7203; PCC9605, *Fischerella* sp. PCC 9605; PCC6912, *Chlorogloeopsis fritschii* PCC 6912.

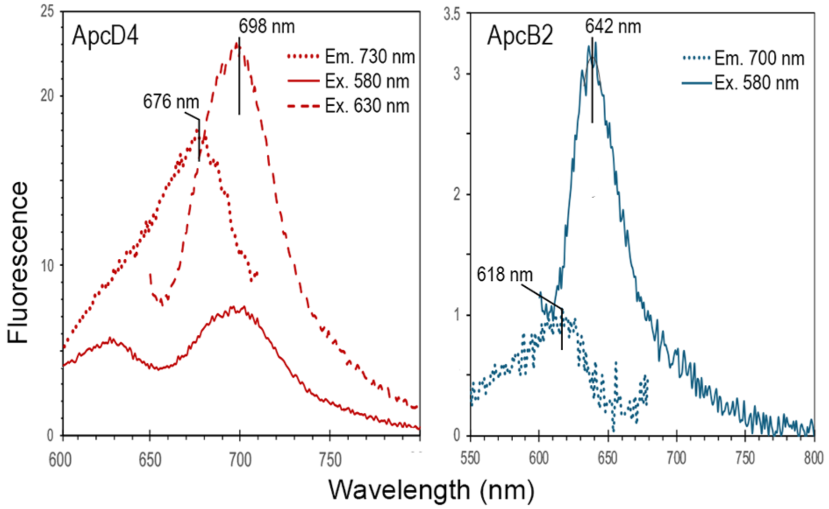

Figure S2. Fluorescence spectra of isolated PCB-ApcD4 and PCB-ApcB2. A. Excitation and emission fluorescence of PCB-ApcD4. Emission fluorescence spectra were recorded using excitation wavelength 580 nm (Solid red line) and 630 nm (dashed red line), respectively. Excitation fluorescence was recorded using emission wavelength of 730 nm (dotted red line). B. Excitation and emission fluorescence of PCB-ApcB2. Emission fluorescence was recorded using excitation wavelength 580 nm (Solid blue line). Excitation fluorescence was recorded using emission wavelength of 700 nm (dotted blue line).

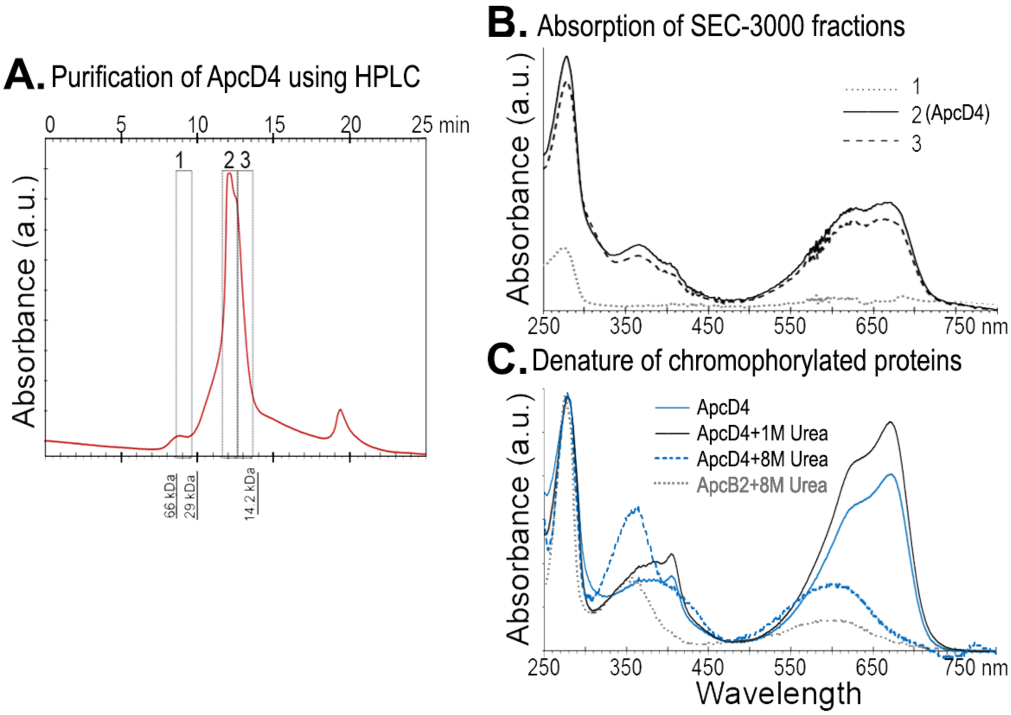

Figure S3. Characterisation of purified PCB-ApcD4 monomer. A. Purification of chromophorylated ApcD4 using BioSep-SEC3000 on uHPLC. The molecular weight markers used for calibration (gel filtration markers kit, Sigma, #MWGF200-1 KT). B. Spectral comparison of resolved 1-3 fractions in buffer containing 40 mM potassium phosphate and 50 mM NaCl, pH 7.4. C. Compered spectral properties of denatured PCB-ApcD4 and PCB-ApcB2 in buffer containing 40 mM potassium phosphate and 50 mM NaCl, pH 7.0 with and without Urea. Black line, initial purified PCB-ApcD4; Solid blue line, PCB-ApcD4 was treated by 1 M urea in the same buffer; Dashed blue line, PCB-ApcD4 was treated by 8 M urea in the same buffer. Grey line, PCB-ApcB2 was treated by 8 M urea in the same buffer. Spectra were normalised at protein bands.

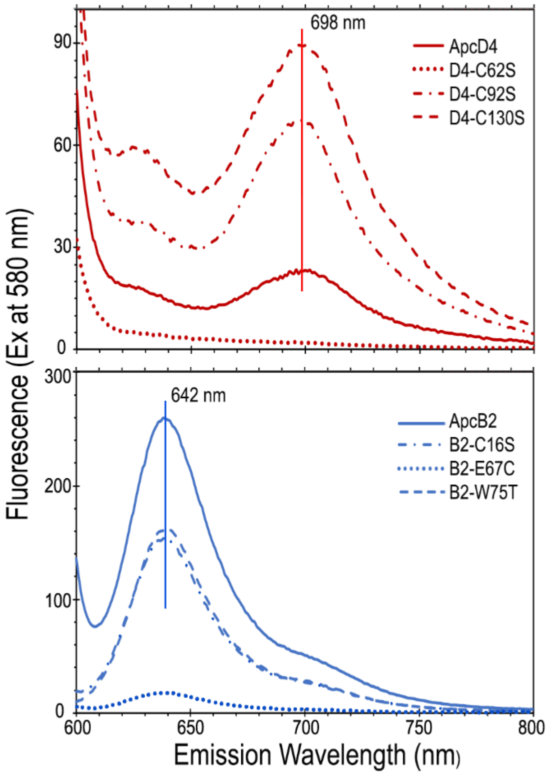

Figure S4. Emission fluorescence comparison. A. Fluorescence recorded using excitation wavelength of 580 nm. Solid red light, PCB-ApcD4; D4-C62S (dotted red line) represents ΔApcD4-C62S; D4-C92S (Dash-dot red line) represents ΔApcD4-C92S; D4-C130S (dashed red line) represents ΔApcD4-C130S. B. Fluorescence recorded using excitation wavelength of 580 nm. Solid blue light, PCB-ApcB2; B2-C16S (dash-dot blue line) represents ΔApcB2-C16S; B2-E67C (dotted blue line) represents ΔApcB2-E67C; B2-W75T (dashed blue line) represents ΔApcB2-W75T.

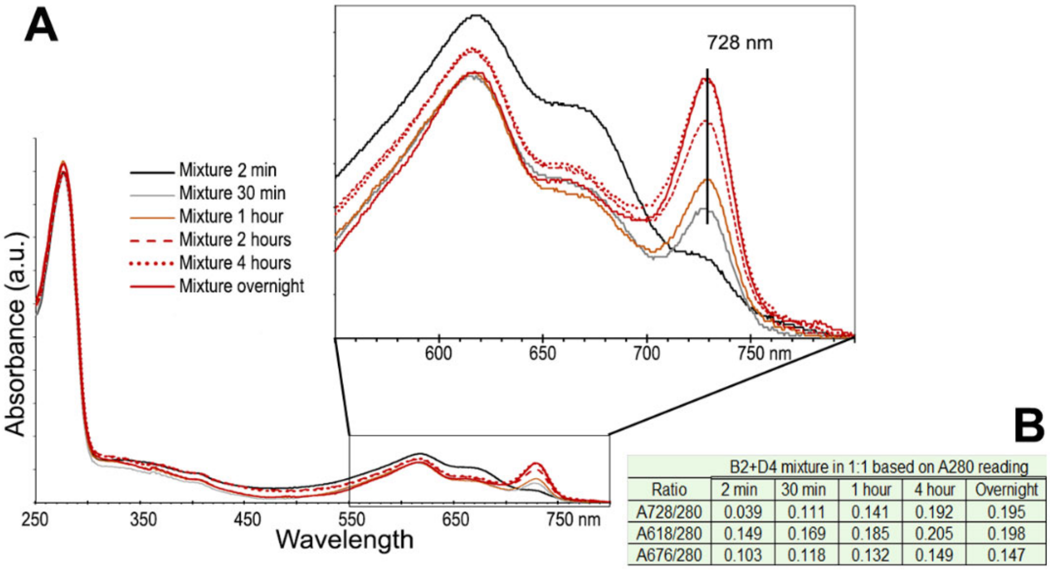

B

B2+D4 mixture in 1:1 based on A280 reading					
Ratio	2 min	30 min	1 hour	4 hour	Overnight
A728/280	0.039	0.111	0.141	0.192	0.195
A618/280	0.149	0.169	0.185	0.205	0.198
A676/280	0.103	0.118	0.132	0.149	0.147

Figure S5. Time course of 728 nm component formation in vitro. The equal amount of isolated PCB-ApcD4 and PCB-ApcB2 were mixed and incubated in dark, rotation in the cold room. A. absorption spectra were recorded at room temperature. B. The ratio of chromophores ($A_{728\ nm}$, $A_{676\ nm}$ and $A_{618\ nm}$) to proteins ($A_{280\ nm}$). The highest A728/A280 of 0.2 was obtained from the overnight sample.

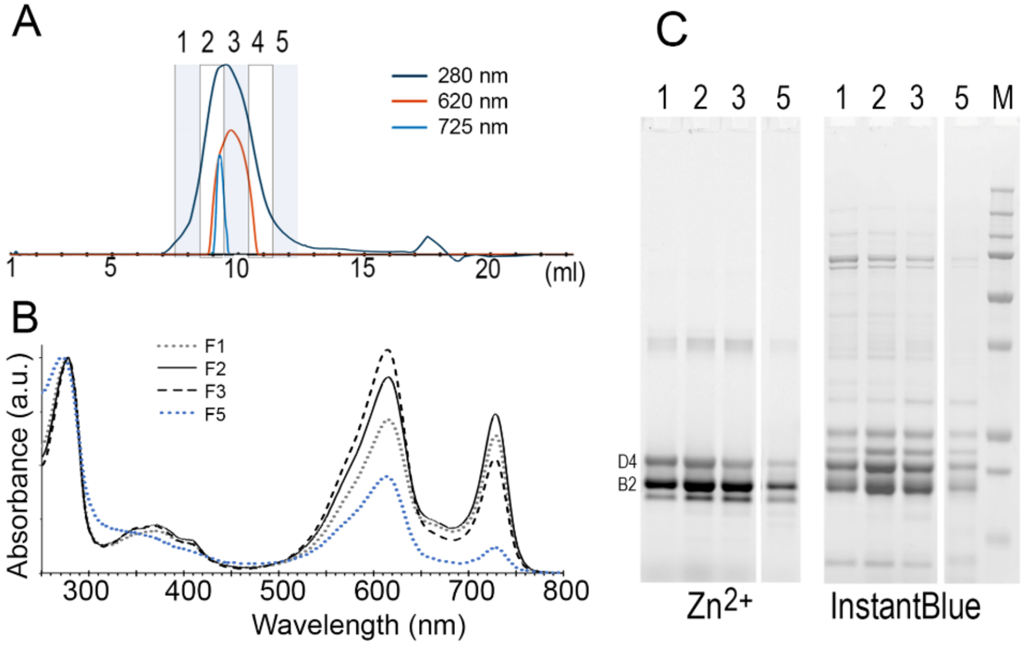

Figure S6. Purification of mixture of PCB-ApcD4 and PCB-ApcB2 using gel filtration column SEC70 (Bio-Rad) on FPLC in buffer containing 40 mM potassium phosphate, 50 mM NaCl, and 5% (w/v) glycerol, pH = 7.4. A. Chromograph profiles detected at 280 nm (dark blue line), 620 nm (red line), and 725 nm (light blue line). Collected fractions are marked as 1-5. B, Compared absorption spectra of collected fractions. C. Protein gels were visualized by Zn^{2+} induced fluorescence and Coomassie Blue (InstantBlue®).

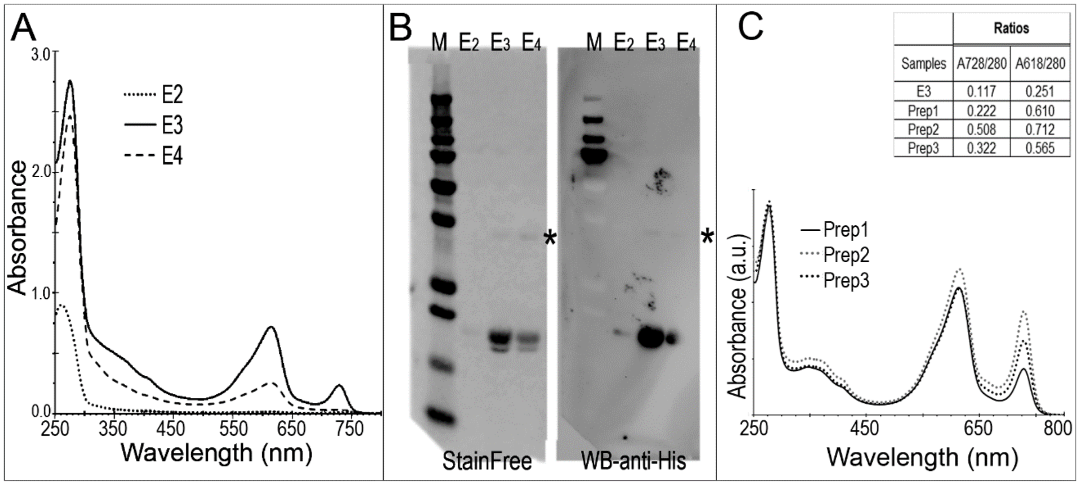

Figure S7. Isolation of APC αβ heterodimer using recombinant His$_6$-ApcB2+ApcD4 heterologous *E. coli* clones. A. Spectra of elution collected from Ni^{2+} affinity chromatography in buffer containing 20 mM potassium phosphate (pH 7.6), 500 mM NaCl, 500 mM imidazole. B. SDS-PAGE and blotting against His-tag, confirming only ApcB2 having His6 target. C. Comparison of three independent preparations (annotated as Prep1, Prep2 and Prep3), indicting different yield level of 728 nm component. The spectra were recorded in desalted buffer of 40 mM potassium phosphate (pH 7.4), 50 mM NaCl. The ratio of bound chromophores to protein was presented as C-insert. D. Protein gels were visualized by Zn^{2+} induced fluorescence and Coomassie Blue (InstantBlue®). M, protein marker; 1,2,3 represent samples of Prep1, Prep2 and Prep3, respectively. 0, represent an empty lane.

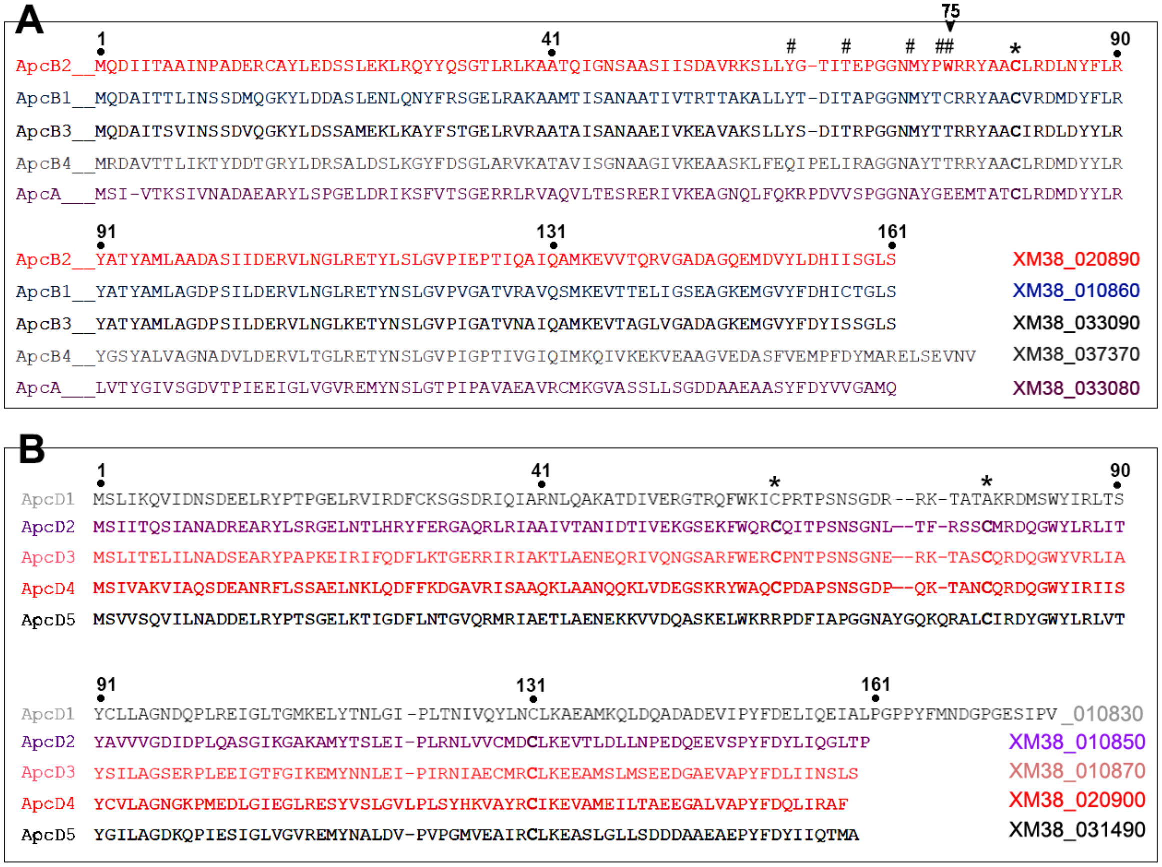

Figure S8. Alignment of allophycocyanin α and β subunit paralogous from *H. hongdechloris*. A. Comparison of ApcBs with ApcA from *H. hongdechloris*. * highlighted Cys residues for PCB binding site. # represents the conserved residues near the PCB of APC α subunits. Trp75 residue of ApcB2 was labelled. B. Comparison of ApcD paralogous in *H. hongdechloris*. * highlighted Cys residues potentially for PCB bindings. Gene IDs were given at the end of the sequences.

Gene ID	Gene names	SAF[1]	APC α subunits
XM38_033080	apcA	1.07	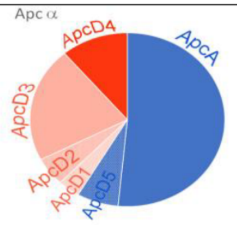
XM38_031490	apcD_5	0.14	
XM38_010830	apcD_1	0.07	
XM38_010850	apcD_2	0.11	
XM38_010870	apcD_3	0.44	
XM38_020900	apcD_4	0.23	
			Apc β subuits
XM38_010860	apcB_1	1.77	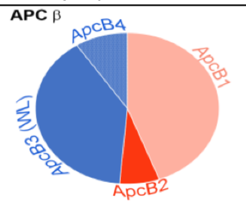
XM38_020890	apcB_2	0.28	
XM38_033090	apcB_3	1.58	
XM38_037370	apcB_4	0.37	

[1] SAF = Detected peptide length/sum of peptide length, %;
Highlight in red, the protein detected from FRL conditions; Highlight in grey, the peptide detected from WL conditions.
Subunits in Blue fonts assemble WL-PBS; subunits in Pink fonts are encoded by FaRLiP gene cluster; the current student subunits are in red fonts, assembling red-shifted PBS.

Figure S9. Proteomic comparison on relative levels of allophycocyanin α and β polypeptides between WL-*H. hongdechloris* and FRL-*H. hongdechloris* (data from Chen *et al.* 2019). ApcD1-D3 and ApcB1 are APC paralogous in FaRLiP gene cluster; ApcB2 and ApcD4 belong to LoLiP gene cluster (current study). ApcA, ApcD5 and ApcB3 are canonical APC subunits.

Supplementary References

Chen, M.; Hernandez-Prieto, M.A.; Loughlin, P.C.; Li, Y.; Willows, R.D. (2019) Genome and proteome of the chlorophyll f-producing cyanobacterium *Halomicronema hongdechloris*: adaptative proteomic shifts under different light conditions. *BMC Genomics* 20:207.

Gisriel CJ, Shen G, Brudvig GW, Bryant DA. (2024b) Structure of the antenna complex expressed during far-red light photoacclimation in *Synechococcus sp*. PCC 7335. *J Biol Chem*. 300:105590. doi: 10.1016/j.jbc.2023.105590.

Appendix (Original gel images)

1. Original gel image for Figures 2, 3 and 5

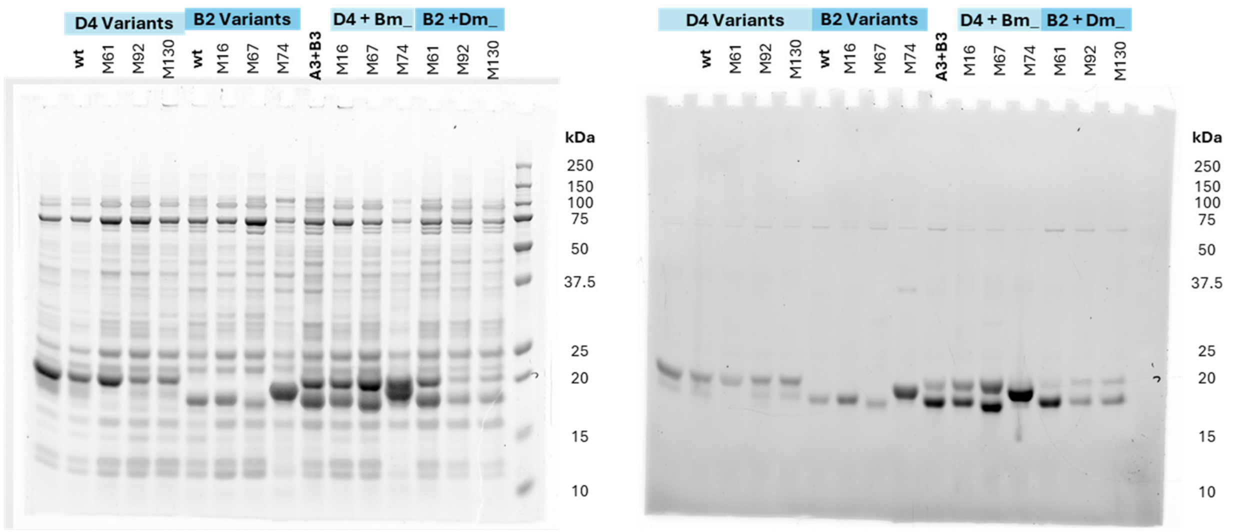

Shake in 50 mM ZnSO4, 30 mins RT, rinse in RO x 2 → Zinc image
Shake in InstantBlue, 30 mins RT, rinse in RO x 2 → Coomassie image

2. Original gel image for Figure 4

1-3: His Column flow through – D4+B2, D4, and B2
5: His-column elution of D4+B2 (individual lysates and co-elution by His-tag column)
6: Fraction collected from SEC70 column (sample for a trial of crystallization)

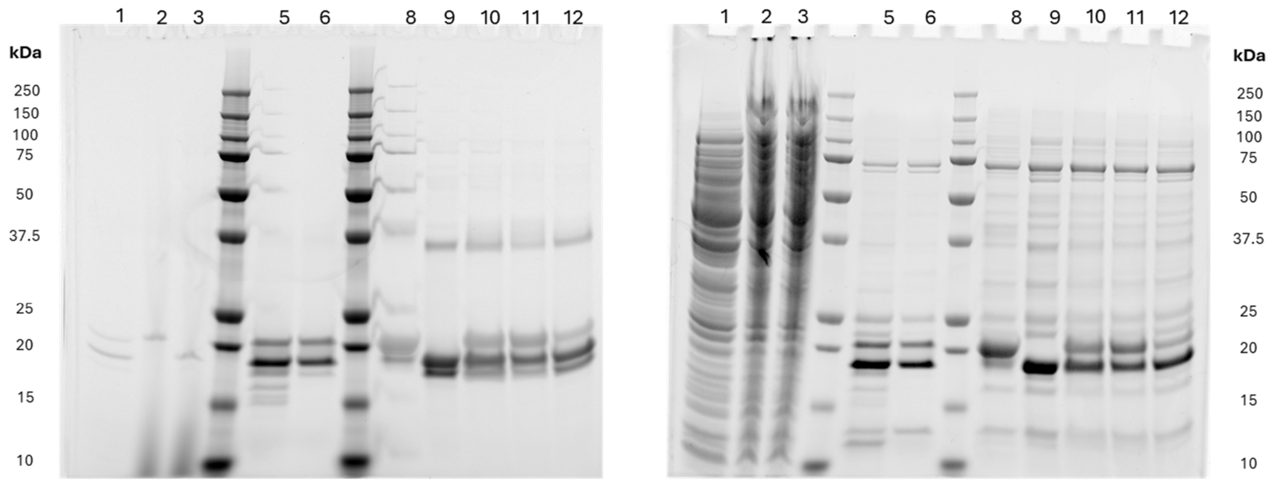

InstantBlue Stain
Tiarne M 2024-04-04 15h36m58s(Coomassie Blue)
